# Universal mental health training for frontline professionals: evaluation of pilot trial in Ukraine

**DOI:** 10.12688/openreseurope.16941.2

**Published:** 2024-06-13

**Authors:** Viktoriia Gorbunova, Vitalii Klymchuk, Nataliia Portnytska, Olha Savychenko, Iryna Tychyna, Georges Steffgen

**Affiliations:** 1 Department of Behavioural and Cognitive Sciences, University of Luxembourg, Esch-sur-Alzette, Luxembourg District, L-4365, Luxembourg; 2 Department of Social Sciences, University of Luxembourg, Esch-sur-Alzette, Luxembourg District, L-4365, Luxembourg; 3 Socio-Psychological Faculty, Zhytomyr Ivan Franko State University, Zhytomyr, Zhytomyr Oblast, 10002, Ukraine

**Keywords:** mental health, mental disorders, task-shifting approach, mental health training, frontline professionals

## Abstract

**Background:**

Increasing accessibility of mental health services and expanding universal health coverage is possible worldwide by using a task-shifting approach as partial delegation of some mental health support tasks to trained non-mental health service providers in order to use the available workforce more efficiently. The Universal Mental Health Training (UMHT), which is dedicated to this aim, was developed and piloted in Ukraine. The UMHT is an educational program for frontline professionals on high-quality and evidence-based responses to the mental health needs of the population they serve.

**Methods:**

The pilot trial of UMHTs’ effectiveness was conducted with 307 frontline professionals divided into 24 training groups. The control group included 211 persons with the same occupation background who participated in training later (waiting list). All the groups took part in eight-hour training, which includes one introductory module that introduces the mental health topic alongside a five-step model of UMHT, two disorders-focused modules with the steps adjusted to work with specific disorders, and the final module that considers possible difficulties frontline professionals might experience. Three effectiveness measurements were used in the outcome assessment: readiness to interact with people with mental health issues at work, mental health awareness and mental health proficiency.

**Results:**

Analysis of the outcome data for the frontline professionals who underwent the UMHT revealed a moderate effect size related to the knowledge of mental health conditions, mental health awareness, and increasing the readiness to interact with people with mental health issues in comparison to the control group.

**Conclusions:**

High-level utilisation of the UMHT at work by trained professionals confirms the effectiveness of the developed intervention. Obtained results favour the continuation of the development of the UMHT and future implementation research in this field in Ukraine and potentially in other low- and middle-income countries.

## Introduction

### Background

Ukraine is a country from the post-Soviet space where psychiatry was a punishment instrument of the regime rather than a health care service ( Frankova *et al.*, 2024; Weissbecker *et al*., 2017). It is the main reason why Ukrainians do not willingly seek psychiatric services and have a lot of stigmatised and self-stigmatised perceptions. For example, one of the most widespread is that mental health difficulties are signs of weakness ( Quirke *et al.,* 2021). Therefore, the task-shifting approach in mental health as a partial distribution of care to frontline professionals seems beneficial for persons with mental health issues and the system itself. It was proven that task shifting in the mental health area eases the workload of health professionals and strengthens community mental health systems, which are essential for low- and middle-income countries ( Javadi *et al.,* 2017; McInnis & Merajver, 2011).

Extending mental health service coverage worldwide is achievable by developing and implementing short, scalable, evidence-based interventions delivered by non-mental health professionals within a task-shifting approach ( Bunn *et al.,* 2021; Paramasivam *et al.,* 2022; Van Ginneken *et al.,* 2011). Numerous interventions have been developed, implemented, and scaled up in recent decades. Most prominent among them are a) the Mental Health Gap Action Programme (mhGAP), which aims to reduce the burden of mental, neurological and substance use disorders through primary mental health intervention by non-specialist health workers ( Chaulagain *et al*., 2020; Keynejad *et al*., 2021) and b) the Mental Health First Aid Programme (MHFA) to support people with mental health problems ( Fuhr *et al*., 2014; Hadlaczky *et al*., 2014; Kitchener & Jorm, 2008). Both offer task shifting, a set of techniques, and an action algorithm for a different mental health condition essential for non-mental health professionals. While the mhGAP is a clinical tool developed mainly for formal healthcare settings (e.g. community and primary health centres), the MHFA is a tool for mental health promotion, stigma reduction and prevention within organisations; it is more suitable for informal workplace implementation.

Also, many mental health training programmes target specific frontline professionals: police ( Fiske *et al*., 2021; Thomas & Watson, 2017; Weaver *et al*., 2022), education workers ( Anderson *et al*., 2019; Whitehurst, 2008), pharmacists ( Frick *et al*., 2021; Samorinha *et al*., 2022) etc.

All the mentioned programmes show evidence of their effectiveness in daily practice, outcomes for the groups of people trainees come into contact with, attitude towards mental health, professional confidence, knowledge, skills, etc. ( Booth *et al*., 2017; Morgan *et al*., 2018; Scantlebury *et al*., 2018; WHO, 2016). Nonetheless, there is a lack of universality (as in the case of the mhGAP), scalability (e.g. local programmes for frontline workers), and feasibility for the LMICs (e.g. the MHFA). Thus, a scalable universal instrument to train frontline professionals to respond to the mental health needs of the specific populations they serve needs to be developed.

This paper aims to analyse the results of a pilot implementation of the Universal Mental Health Training (UMHT) using a quasi-experimental plan with experimental and control groups without prior randomisation due to real-life restrictions (the budget limitations, the proximity of some pilot regions to the war demarcation line, professionals' work overload, and the priority of implementation over research needs). Three effectiveness measurements were used in the outcome assessment: readiness to interact with people with mental health issues at work, mental health awareness and mental health proficiency.

### Universal Mental Health Training (UMHT): a new instrument for informal mental health response by frontline professionals

The UMHT is an educational program developed to train frontline professionals on high-quality and evidence-based responses to the mental health needs of the population they serve. Police officers, emergency responders, social services workers, educators, pharmacists, priests, and other professionals interact with many people daily. Whereas their professional roles imply working with people in crisis who experience strong emotions and require support, a high level of mental health awareness and skills to manage mental health issues are needed. Therefore, UMHT was developed as an educational instrument for Ukrainian frontline professionals to raise their mental health awareness, reduce stigma toward people with mental disorders and develop particular skills for giving support.

The training is called Universal because its 5-step model offers a standard frame for interaction with people with mental health issues. Also, it is Universal because it is suitable for different types of frontline workers – the general interaction structure is not changing, only the set of relevant mental health conditions.

***Mental health disorders covered by UMHT.*** UMHT covers the 18 most prevalent mental health disorders throughout lifespan development, which are defined according to DSM-5: depressive disorder, intellectual disability, panic disorder, post-traumatic/acute stress disorder, attention-deficit/hyperactivity disorder, social anxiety disorder, disruptive, impulse-control, and conduct disorders, autism spectrum disorder, delirium, separation anxiety disorder, specific phobias and agoraphobia, illness anxiety disorder, feeding and eating disorders, elimination disorders, sleep-wake disorders, substance-related disorders, gambling disorder, neurocognitive disorders ( APA, 2013).

***UMHT target audience***. The target audience for the UMHT delivery is frontline professionals (workers), defined as professionals whose jobs involve close personal communication with people (clients, service users, etc.) ( Blau *et al*., 2021). The most common types of frontline professionals have been identified according to Ukrainian occupational regulations and standards: social workers, educators, police officers, priests and clerics, military volunteers, workers of occupation centres, emergency workers, etc. ( Derzhspozhyvstandart of Ukraine, 2010).

***Universal 5-step response model***. The mhGAP and the MHFA have become a methodological base for the UMHT: in each case, it offers a 5-step model that is in tune with the MHFA action plan and mhGAP sections. The UMHT steps go one by one as a chain of action (recognise mental health conditions, validate a condition with a person, give support, refer for professional help, ensure that professional service is received). The mhGAP sections have the same step-by-step logic and lead a helper from assessment through management to follow-up. In contrast, the MHFA steps can be done in any order (approach and assess for risk of suicide or harm, listen nonjudgmentally, give reassurance and information, encourage appropriate professional help, encourage self-help and other support strategies) ( Kitchener & Jorm, 2008; WHO, 2016).

**Figure 1.**
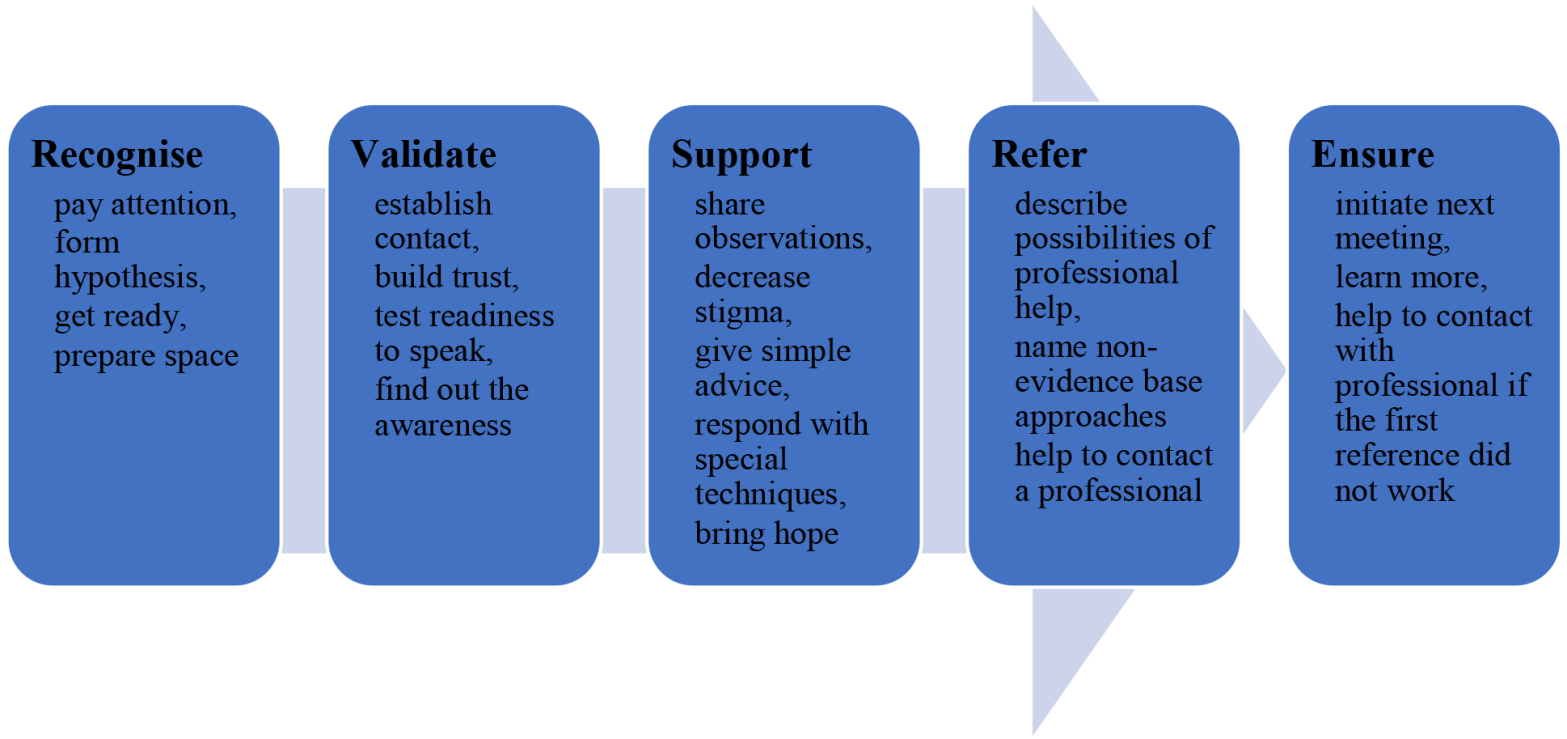
5-step model of UMHT.

The name of every UMHT step reflects its aim and implies the set of necessary actions ( Figure 1.). Step 1 aims to **recognise** mental health conditions. It means a helper should pay attention to persons, their behaviour, reactions, communication, etc. Then, it is time for a hypothesis about a possible mental health condition and the types of support that person needs. The following action is to prepare for the conversation, plan it, and prepare themselves emotionally. Finally, the helper should prepare space for interaction.

Step 2 aims to **validate** the person’s condition. It is the step for initiating the conversation, building trust, testing the readiness to speak, and finding out the level of awareness of the person in their condition.

Step 3 is about providing **support** - sharing observations, decreasing stigma, giving simple advice, responding with special techniques, and instilling hope.

Step 4 aims to provide relevant **references** if there is a need. At this step, the frontline professional describes possibilities of professional help, names non-evidence-based approaches, and helps contact a professional.

Finally, in step 5, there is an aim to **ensure** that person received support or followed the advice etc.: initiate the next meeting, learn more, and help to contact with professional if the first reference did not work.

The steps and actions are adjusted for every mental condition. All the nuances are highlighted on the appropriate step with the support of the evidence-based recommendation, the primary sources for which were NICE guidelines ^1^.

It is foreseen that those steps will be delivered in sequence to the service users, clients etc., by frontline professionals within their working frames.

***UMHT dissemination mode***. At this stage of development, the UMHT is disseminated by the Training of Trainers (ToT) approach, highlighted in the mhGAP Operation Guide ( WHO, 2018). The mentioned approach implies engaging high-level experts to coach trainers who are less experienced in a field. In this way, a ToT scheme builds a pool of competent trainers to disseminate skills and knowledge. According to such requirements, the UMHT developers conduct Training of Trainers and supervise participants while they deliver the training to specific groups of frontline professionals.

This pilot research's objective is to evaluate the UMHT delivery outcomes. The extended description of the standard mode of the training delivery to the frontline professionals by trained trainers is provided below.

**Target audience** (type of frontline professionals) selection is the first step for the UMHT delivery. After the audience selection and understanding of the subject and specifics of interaction with service users, it is essential to make more **inquire** about professional interaction. Age, occupation, a typical way of communication, widespread problems, mental health difficulties and other features of people with whom frontline professionals work are essential for the next step – **selecting** the mental health conditions for inclusion in the training program. Also, **combining** the pre-defined proposals and requests from the professionals is important. Every target audience needs a particular set of mental conditions to be included in the program. Therefore, the design of each training follows these needs. Also, important to know the time professionals can dedicate to participating in the training.

Each training **delivery** consists of compulsory modules (Introductory module and Final module) and selective modules, each dedicated to specific mental health conditions. Each module lasts 90 minutes and includes the standard set of slides, examples, role-play exercises and discussions. Usually, 2–4 conditions are selected for the training delivery (see example in Table 1). After the training delivery, all the participants have the possibility of **supervision support**. At the supervision meetings, participants discuss particular cases, problems, and concerns related to their practice in real situations. Such case consultations can be provided individually or in small groups to enable mutual learning and experience exchange.

**Table 1.** Training modules allocation.

Disorders-focused modules included in the group-specific curriculum	Number of training groups						
	Social workers (12)	Educators (4)	Police officers (4)	Priests and clerics (1)	Military volunteers (1)	Workers of occupation centres (1)	Emergency workers (1)
Introductory module	12	4	4	1	1	1	1
Depressive disorders	12						
Intellectual disabilities		1					
Panic disorder	9						
Post-traumatic / acute stress disorder	3		2	1	1	1	1
Attention-deficit / hyperactivity disorder		2					
Social anxiety disorder				1		1	
Disruptive, impulse-control, and conduct disorders			3		1		1
Autism spectrum disorder		5	1				
Delirium			2				
Final module	12	4	4	1	1	1	1

**Pre- and post-training assessment** is a part of the training delivery. It includes a set of questionnaires described in the section “Methods”.

## Methods

### Participants

The pilot trial of UMHTs’ effectiveness was conducted with 307 frontline professionals divided into 24 training groups (social workers (12 groups, 128 persons), educators (4, 63), police officers (4, 60), priests and clerics (1, 15), military volunteers (1, 12), workers of occupation centres (1, 13), emergency workers (1, 16)). All participants were recruited for training by their team leaders, who were informed about training possibilities by letters sent from the training developers. The only requirement for participation was working in the field with people. No incentives were proposed to participants.

The control group included 211 persons with the same occupation background who participated in training later (waiting list). The control group consisted of social workers (97 persons), educators (32), police officers (40), priests and clerics (12), military volunteers (13), workers of occupation centres (7), and emergency workers (10).

### Training delivery

All the groups took part in 8-hour training which includes one introductory module that introduces the mental health topic alongside with 5-step model, two disorders-focused modules with the steps adjusted to work with specific disorders and the final module that considers possible difficulties and ways of their solving. Every group studied a particular set of disorders that depended on their target audiences’ needs. The pilot trial included such disorders as depressive disorders (included in the training curriculum for 12 groups), intellectual disabilities (1), panic disorder (9), post-traumatic/acute stress disorder (9), attention-deficit/hyperactivity disorder (2), social anxiety disorder (2), disruptive, impulse-control, and conduct disorders (5), autism spectrum disorder (6), delirium (2). The detailed allocation of the modules is present in Table 1.

All the trainings were carried out between February and December 2021.

### Measurements

Three effectiveness measurements were used in the outcome assessment. Personal data, except occupation, were not collected. E-mails were gathered separately to send a reminder for a one-month follow-up test.

***Readiness to interact with people with mental health issues at work***. To measure the changes in readiness to interact with people with mental health issues at work (according to the 5-step model), all participants self-assessed their general readiness as well as readiness to do particular actions according to the 5-step model on a five-point scale (from 5 - “absolutely ready” to 1 - “absolutely not ready”).

In the instruction, participants were asked: “Reading the next statements, please assess your readiness for a different kind of interaction with people with mental health conditions. The scale is from 1 to 5, where 1 is the absolute absence of readiness, and 5 – is the absolute readiness”.

The next set of statements was proposed to participants:

– Readiness to interact with people with mental health issues at work (general readiness).
– Readiness to recognise mental health conditions (readiness for step 1 of the 5-step model).
– Readiness to initiate and lead conversation with a person with mental health issues and his/her caregivers (readiness for step 2).
– Readiness to support a person with mental health issues and his/her caregivers (readiness for step 3).
– Readiness to refer a person with mental health issues, and his/her caregivers, to professional support (readiness for step 4).
– Readiness to ensure that professional help is received by a person with mental health issues and his/her caregivers (readiness for step 5).

***Mental health awareness***. Mental health awareness assessment was based on the KAP (knowledge, attitudes, and practices) model ( Andrade *et al*., 2020) *.* There is the experience of using such KAP-based surveys in Ukraine ( Quirke *et al*., 2021) *.* Based on the KAP model, a short survey was developed related to the knowledge about mental health issues, attitudes toward people with mental health disorders, and practice of interaction with them.

Knowledge regarding people with mental disorders was assessed with the query: “Choose the statements that apply to people with mental health disorders” (max = 8 scores, where each score was awarded either for a choice of a correct statement or for a non-selection of a wrong statement):

– They are dangerous to the people around them.
– They are themselves guilty of their condition.
– They are incapable of true friendships.
– They can work.
– By appearance, it is clear that the person is not all right.
– Anyone can have a mental disorder.
– Mental disorders are incurable.
– Most people with mental disorders can recover.

Attitude towards people with mental issues was assessed with the question: “What is the best way of behaviour for people with mental health issues?” (max = 8 scores):

– Do not tell anyone about their condition.
– Discuss everything with a doctor, but do not inform relatives.
– Hide this information at work/school.
– Tell loved ones and ask for help from specialists.
– Hide it from the family.
– Live among those like themselves.
– Should not marry and have children.

The question for assessment practices of interactions with people with mental disorders: “What is the proper way of interactions with people with mental health disorders?” (max = 9 scores):

– You would better avoid any contact with them.
– You shouldn’t allow them to make any decisions.
– You would better avoid working with them in one team or performing tasks together.
– You should be careful about conversations with them.
– You should be ashamed and try to hide the fact you have a relative with a mental health disorder.
– They should have the same rights as anyone else.
– It is normal to have a friend with a mental health disorder.
– It is normal to marry a person with a mental health disorder.
– You should treat them with care and sympathy.

Practices of care about people with mental health issues were analysed with the question: “What is the best way to care about people with mental health issues?” (max = 6 scores):

– In a psychiatric hospital where they are under supervision and control (psychiatrist).
– Outside the hospital in specialised centres or privately (psychologist, psychotherapist).
– Alternative methods of treatment (traditional medicine, homoeopathy, vitamins, massage).
– Normal family relationships is the best treatment.
– Do not waste energy, it is not possible to cure mental disorders.
– At the primary level of health care (family doctor, paediatrician, general practitioner).

Mental health awareness scores were collected as the sum of scores for each scale.

***Mental health proficiency***. Mental health proficiency, as the ability to recognise mental health disorders’ symptoms, was assessed by the tests that include correct and non-correct symptoms. Three true and two false symptoms (based on DSM-5) were offered for selection in each case. Mental health proficiency was estimated as the sum of the correct choices of symptoms for every disorder learned by participants. For instance, the participants who worked during the training with depressive disorders should choose all appropriate parameters among depressed mood, markedly diminished interest or pleasure in almost all activities, excessive or inappropriate feelings of worthlessness or guilt, inattention as, difficulties following instructions and failure to finish tasks, restlessness as fidgeting with or tapping hands or feet or squirming in the seat.

***Additional one-month follow-up questions***. Additional questions for the one-month follow-up test were: “Did you work after the training with people with mental health issues that you studied?”, “What kind of the issues?”, “Did you use training knowledge and skills?”, “Which knowledge and skills did you use in particular?”

### Data collection

Participation in data collection was voluntary and not coerced by employers or researchers. Participants were free to refuse to enter their answers; all questionnaires were anonymised. Data were collected in paper forms before and after the training sessions. After converting the data into electronic format, the questionnaires' paper forms were destroyed. Storage of the sensitive data was conducted in adherence to GDPR. Email addresses were collected separately for the purpose of an invitation to follow-up tests, after which they were deleted.

### Data analysis

Descriptive statistics (mean, standard deviation) were used to describe the general results. Student’s T-test for independent samples was used to compare independent samples (pre-test, control and training groups). Student’s T-test, dependent samples, was used to analyse the statistical significance of changes before and after the training and one month after. Cohen’s effect size coefficients were calculated to estimate the size of the effect. Two-way ANOVA was utilised to analyse the significance of differences for aggregated indexes in training and control groups before and one month after the training.

### Study design

The study was quasi-experimental (no complete randomization was possible at this piloting stage). Two groups were involved, the experimental group (received UMHT) and the control group (no training, waiting list). The complete outline of the study design is in Table 2.

**Table 2.** Outline of the study design.

Activity	Measures	Groups	
		Experimental	Control
**Pre-test** (before the training)	Readiness to face people with mental health issues at work	X	X
	Mental health awareness	X	X
	Mental health proficiency	X	n/a ^2^
	Additional one-month follow-up questions	n/a	n/a
**Intervention (UMHT)**		X	
**Post-test** (immediately after the training)	Readiness to Face People with Mental Health Issues at Work	X	n/a
	Mental health awareness	X	n/a
	Mental health proficiency	X	n/a
	Additional one-month follow-up questions	n/a	n/a
**One month follow up test** ** / re-test**	Readiness to Face People with Mental Health Issues at Work	X	X
	Mental health awareness	X	X
	Mental health proficiency	X	n/a
	Additional one-month follow-up questions	X	n/a

^2^n/a – not applicable

Answers from only 238 persons from the training group who passed all three rounds of questionnaires (pre-test, post-training-test, and one-month follow-up test) were considered for analysis: social workers (94 persons), educators (54), police officers (43), priests and clerics (12), military volunteers (12), workers of occupation centres (10), emergency workers (14). All data are available in Zenodo ( Gorbunova & Klymchuk, 2023).

## Results

### Statistical comparison between the training and the control group before the training

Training and control groups were compared to check the pre-intervention differences that could interfere with the study results. Comparison data is available in Table 3.

**Table 3.** Training and control group comparison.

	Pre-test (±SD)		P-value ^3^
	Training group	Control group	
**Readiness to face people with mental health issues at work**			
Readiness to face people with mental health issues at work	3.28 (1.07)	3.64 (0.99)	**-3.70** **Р <0.01**
Readiness to recognise MH condition	3.15 (1.04)	3.65 (0.94)	**-5.35** **Р <0.01**
Readiness to validate a condition with a person	3.23 (1.12)	3.66 (1.09)	**-4.12** **Р <0.01**
Readiness to give support	3.49 (1.13)	4.03 (0.89)	**-5.65** **Р <0.01**
Readiness to refer for professional help	3.60 (1.16)	4.17 (0.95)	**-5.72** **Р <0.01**
Readiness to ensure that professional help is received	3.59 (1.16)	4.00 (0.99)	**-4.04** **Р <0.01**
**Mental health awareness**			
Knowledge regarding people with mental issues: “Choose the statements that apply to people with mental health issues”. (max = 8 scores)	5.76 (1.32)	6.04 (1.47)	**-2.11** **Р <0.05**
Attitude towards people with mental issues: “What is the best way of behaviour for people with mental health issues?” (max = 7 scores)	6.81 (0.49)	6.82 (0.50)	-0.21 P <0.25
Practices of interactions with people with mental issues: “What is the proper way of interactions with people with mental health issues?” (max = 9 scores)	6.82 (1.40)	6.89 (1.63)	-0.49 P <0.25
Practices of care about people with mental health issues: What is the best way of helping people with mental health issues?” (max = 6 scores)	3.58 (0.93)	3.73 (1.02)	-1.62 P <0.07

^3^Student’s t-test, independent samlpes

Two parameters were assessed both in the training and control group. There were small but significant differences in the readiness to face people with mental health issues at work, with a slightly higher level for the control group. Within the mental health awareness, only one scale was different, the knowledge scale, which was higher in the control group.

### Changes in the training group over time

***Readiness to face people with mental health issues at work***. Data about changes in readiness to face people with mental health issues at work for the training groups are available in Table 4. It shows the mean score for every scale with its original name.

**Table 4.** Changes in readiness to interact with people with mental health issues at work.

	Pre-test (±SD)	Post-test (±SD)	One- month follow-up test (±SD)	P-value ^4^		Effect size	
				pre-test vs post- test	pre-test vs one-month follow-up test	pre-test vs post-test	pre-test vs one-month follow-up test
Readiness to face people with mental health issues at work	3.28 (1.07)	3.78 (0.90)	3.68 (0.85)	**4.74** **Р <0.001**	**3.59** **р <0.001**	0.51	0.41
Readiness to recognise MH condition	3.15 (1.04)	3.91 (0.78)	3.64 (0.83)	**9.37** **р <0.001**	**5.83** **р <0.001**	0.83	0.52
Readiness to validate a condition with a person	3.23 (1.12)	3.92 (0.93)	3.69 (1.02)	**7.64** **р <0.001**	**4.97** **р <0.001**	0.67	0.43
Readiness to give support	3.49 (1.13)	4.10 (0.85)	3.97 (0.93)	**6.88** **р <0.001**	**5.25** **р <0.001**	0.61	0.46
Readiness to refer for professional help	3.60 (1.16)	4.12 (0.88)	4.06 (1)	**5.73** **р <0.001**	**4.78** **р <0.001**	0.51	0.42
Readiness to ensure that professional help is received	3.59 (1.16)	4.09 (0.86)	4.00 (1.03)	**5.64** **Р <0.001**	**4.34** **Р <0.001**	0.49	0.37

^4^Student’s t-test

There were significant changes (р <0.001) in the readiness to face people with mental health issues in general and to interact with them at every step of mental support. We can see a minor drop in mean readiness scores between post-training and one-month follow-up tests; however, it doesn’t affect the p-value.

The effect size, calculated for every step of support provided, is in the range from medium to large, with the lowest level for the “Readiness to ensure that professional help is received” (0.49; pre-test vs post-test) and the largest for the “Readiness to recognise MH condition” (0.83; pre-test vs post-test). After the one-month, the effect size decreased to the under-medium level for all steps.

***Mental health awareness***. The mean scores and estimations of the significance of differences for pre-, post-, and one-month follow-up tests for every scale are shown in Table 5.

**Table 5.** Changes in mental health awareness in the training group.

	Scores for correct choices			P-value ^5^		Effect size	
	pre-test (±SD)	pre-test vs post- test (±SD)	pre-test vs one-month follow-up test (±SD)	pre-test vs post- test	pre-test vs one-month follow-up test	pre-test vs post- test	pre-test vs one-month follow-up test
Knowledge regarding people with mental issues: “Choose the statements that apply to people with mental health issues”. (max = 8 scores)	5.76 (1.32)	5.63 (1.60)	6.38 (1.58)	-1.06 p = 0.29	**4.95** **р <0.001**	-0.09	0.43
Attitude towards people with mental issues: “What is the best way of behaviour for people with mental health issues?” (max = 7 scores)	6.81 (0.49)	6.72 (0.65)	6.86 (0.44)	-1.80 p = 0.72	1.45 р = 0.14	-0.16	0.11
Practices of interactions with people with mental issues: “What is the proper way of interactions with people with mental health issues?” (max = 9 scores)	6.82 (1.40)	6.95 (1.45)	7.42 (1.43)	1.056 p = 0.29	**4.89** **р <0.001**	0.09	0.42
Practices of care about people with mental health issues: What is the best way of helping people with mental health issues?” (max = 6 scores)	3.58 (0.93)	3.51 (1.01)	4.05 (0.89)	-0.78 Р = 0.44	**6.014** **р <0.001**	0.07	0.52

^5^Student’s t-test

No significant changes due to the mental health-related knowledge, attitude and behaviour happened immediately after the training. But after the one-month follow-up assessment, the differences increased and became statistically significant for four scales (р <0.001). The Attitude scale (р = 0.14) is the only one that doesn’t change. The level of effect size is medium or under-medium for all significant changes.

***Mental health proficiency***. Table 6 presents mean scores for all disorders studied by training participants. The level of p-value is significant in both cases of comparison. These data were collected only for the training group because of the need to measure changes in the understanding of disorders studied by training participants.

**Table 6.** Changes in mental health proficiency for the training group.

	Scores for true answers			P-value ^6^		Effect size	
	pre-test (±SD)	post-test (±SD)	one-month follow-up test (±SD)	pre-test vs post-test	pre-test vs one-month follow-up test	pre-test vs post- test	pre-test vs one- month follow-up test
Recognition of mental disorders’ symptoms (max = 5 scores)	3.38 (1.07)	3.60 (1.03)	3.75 (0.95)	3.69 р <0.001	6.24 р <0.001	-0.21	-0.37

^6^Student’s t-test

**Figure 2.**
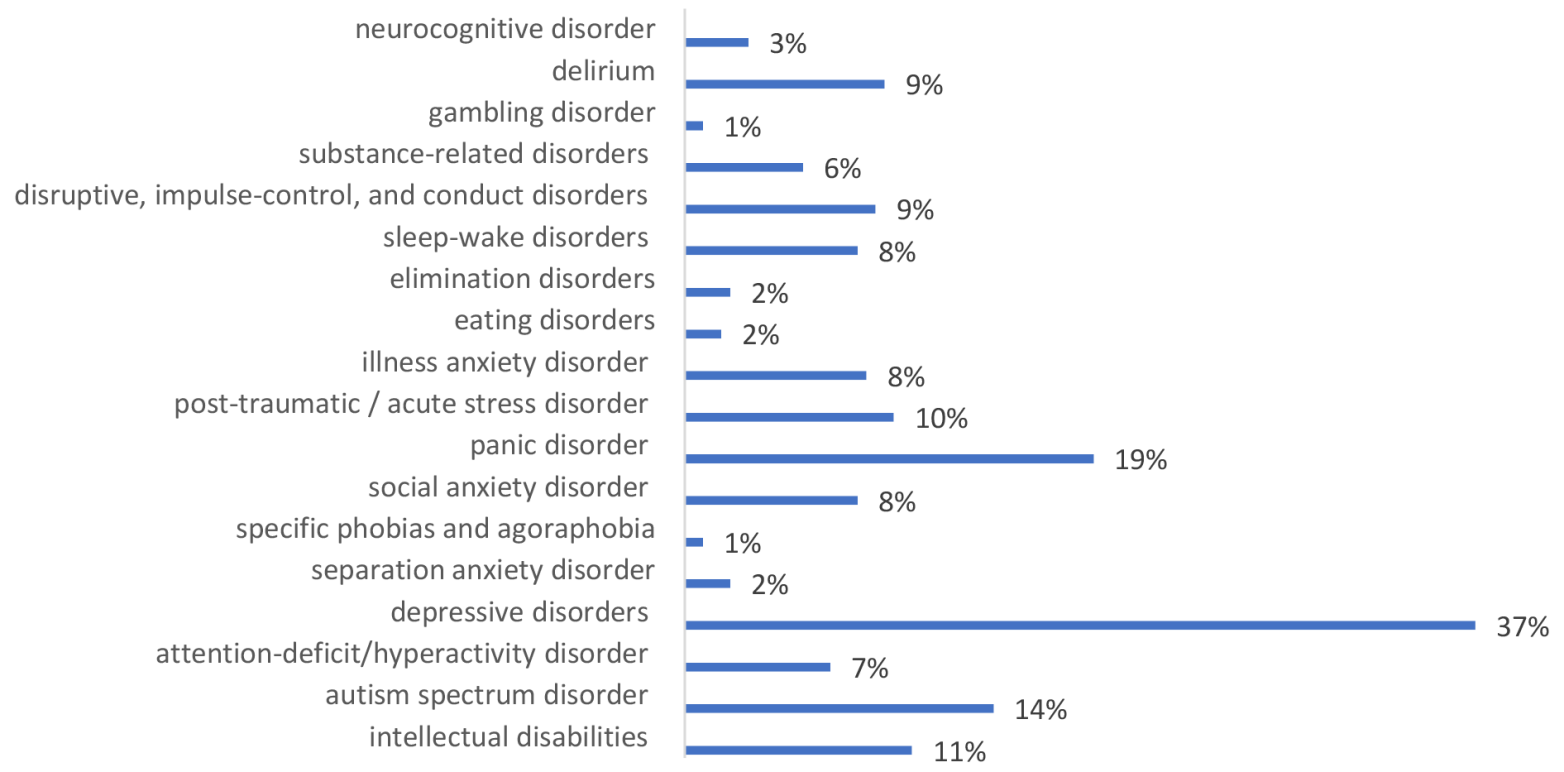
Mental health conditions that training participants encountered in people they worked with after the training.

The additional questions reveal participants’ estimation of the professional usefulness of training knowledge and skills. 56.6% of participants (133 from 238 persons), during the month after the training, worked with people with mental health conditions learned in training. Most often, they met people with depressive disorders (37%), panic disorder (19%) and autism spectrum disorder (14%) ( Figure 2.).

**Figure 3.**
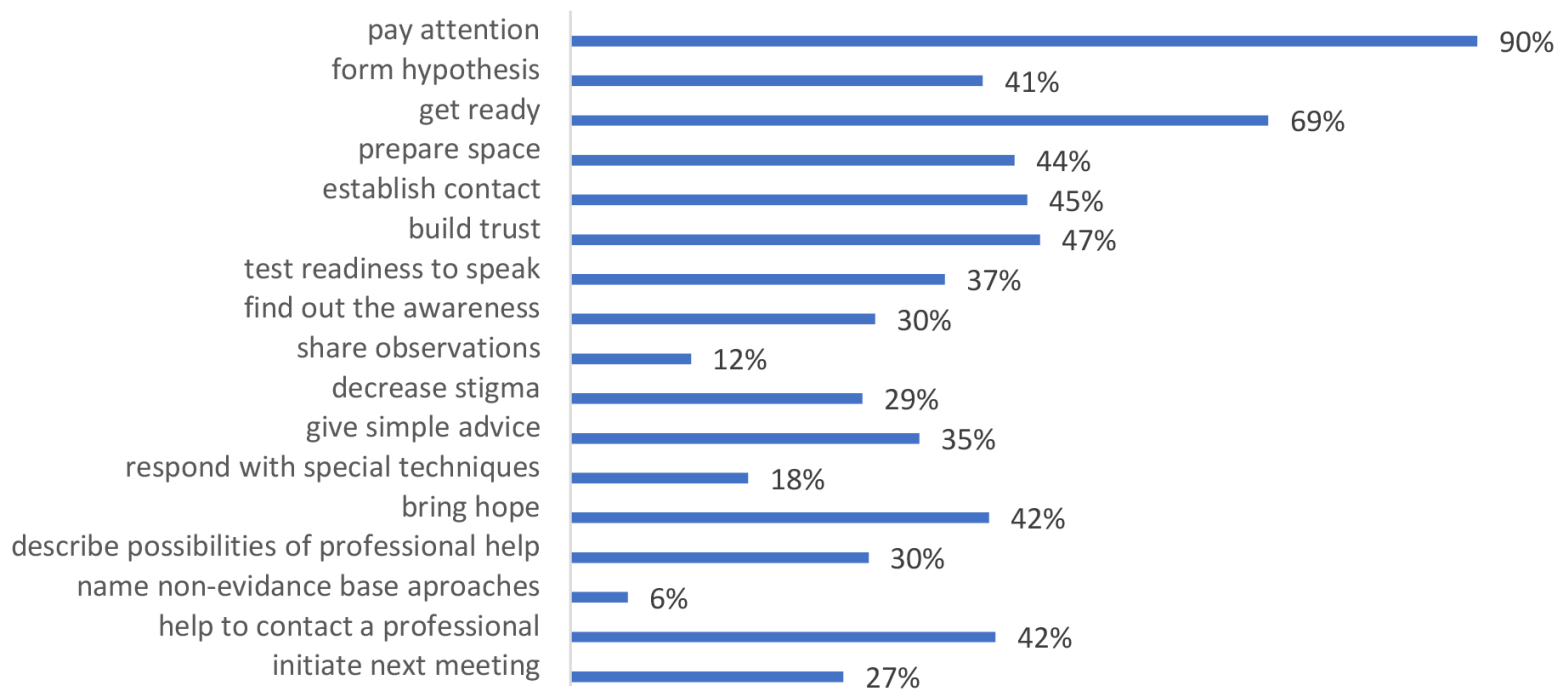
Training knowledge and skills that training participants used after the training.

Figure 3 shows assessment data of frequency for using knowledge and skills by participants during the month following training. 67.7% of participants (159 from 238 persons) noticed that they used trained knowledge and skills in the following period. The leading skills were paying attention to the person’s condition (90%) and getting ready for conversation (69%).

### Changes in the control group over time

***Readiness to face people with mental health issues at work***. Data for the control group is present in Table 7.

**Table 7.** Changes in readiness to interact with people with mental health issues at work (control group).

	Test (±SD)	One-month retest (±SD)	The P-value ^7^ for test vs one-month retest	The effect size for test vs one-month retest
Readiness to face people with mental health issues at work	3.64 (0.99)	3.39 (0.98)	-2.47 Р <0.05	-0.25
Readiness to recognise MH condition	3.65 (0.94)	3.38 (0.99)	-2.80 Р = 0.05	-0.28
Readiness to validate a condition with a person	3.66 (1.09)	3.49 (1.13)	-1.57 Р = 0.117	-0.15
Readiness to give support	4.03 (0.89)	3.79 (1.06)	-2.41 Р = 0.016	-0.25
Readiness to refer for professional help	4.17 (0.95)	3.93 (1.09)	-2.30 Р = 0.021	-0.23
Readiness to ensure that professional help is received	4.00 (0.99)	3.87 (1.11)	-1.27 Р = 0.205	-0.12

^7^Student’s t-test

Notably, control group participants have no increases in the general readiness to interact with people with mental health issues at work. Otherwise, it seems that without training and supervision, frontline professionals lose their previous willingness to face people with mental difficulties, recognise mental health conditions, give support and refer them for professional help.

***Mental health awareness***. Table 8 shows the data without any sufficient changes for the control group.

**Table 8.** Changes in mental health awareness (control group).

	Test (±SD)	One-month retest (±SD)	The P-value ^8^ for test vs one- month retest	The effect size for test vs one- month retest
Knowledge regarding people with mental issues: “Choose the statements that apply to people with mental health issues” (max = 8 scores)	6.04 (1.47)	5.77 (1.52)	-1.78 Р = 0.075	-0.18
Attitude towards people with mental issues: “What is the best way of behaviour for people with mental health issues?” (max = 7 scores)	6.82 (0.50)	6.89 (0.36)	1.34 Р = 0.181	0.16
Practices of interactions with people with mental issues: “What is the proper way of interactions with people with mental health issues?” (max = 9 scores)	6.89 (1.63)	6.66 (1.18)	-1.51 Р = 0.130	-0.16
Practices of care about people with mental health issues: What is the best way of helping people with mental health issues?” (max = 6 scores)	3.73 (1.02)	3.61 (0.99)	-1.30 Р = 0.194	-0.12

^8^Student’s t-test

The absence of significant changes in the control group data over time in the presence of the changes in the training group confirms the hypothesis about UMHT as the reason for such changes, despite the initial difference in the groups. The scores in the control group were higher, but in the absence of the UMHT, they remained the same, whereas the scores of the training group grew significantly.

### Results of the ANOVA for aggregated indexes

Additional two-way ANOVA was conducted to test the hypothesis of the significance of the impact of the UMHT training on the readiness of trainees to interact with people with mental health issues and on their mental health awareness. Aggregated indexes (average values) were calculated for two measures – “Readiness to interact with people with mental health issues at work” and “Mental health awareness” ( Table 9).

**Table 9.** Aggregated indexes (average values) for the control and training group (pre-test and 1-month follow-up test).

N	Group	Pre-test (±SD)	1-month follow up test (±SD)
**Readiness to interact with** ** people with mental health issues at work**			
238	Training group	3.39 (1.11)	3.84 (0.96)
211	Control group	3.86 (0.98)	3.71 (1.06)
**Mental health awareness**			
238	Training group	5.74 (1.07)	6.18 (1.09)
211	Control group	5.58 (1.16)	5.73 (1.01)

***ANOVA for the “Readiness to interact with people with mental health issues at work”***. Two independent factors (variables) were considered for the analysis with two levels for each of them – Test (pre-test vs 1-month follow-up test) and Group (training vs control). Aggregated measure *“Readiness to interact with people with mental health issues at work”* was assigned as the dependent variable. Results of the ANOVA are shown in Table 10.

**Table 10.** Readiness to interact with people with MH issues at work (ANOVA results).

Source	Type III Sum of Squares	df	Mean Square	F	Sig.
Corrected Model	32.117 ^a^	3	10.706	10.093	<0.001
Intercept	12248.856	1	12248.856	11547.733	0.000
**Group (T, C)**	6.375	1	6.375	6.010	**0.014**
**Test (Pre, 1-month)**	4.535	1	4.535	4.276	**0.039**
Group * Test	19.970	1	19.970	18.826	**<0.001**
Error	948.279	894	1.061		
Total	13240.000	898			
Corrected Total	980.396	897			

a) R Squared = 0.033 (Adjusted R Squared = 0.030)

**Figure 4.**
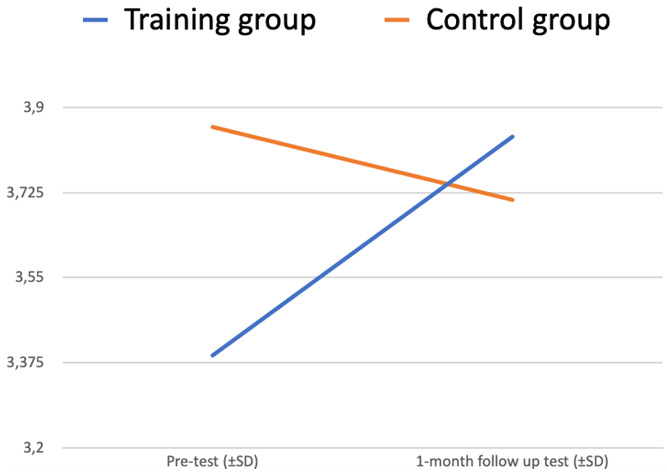
Readiness to interact with people with MH issues at work (ANOVA plot).

There is a statistically significant interaction between two factors – Test (pre-test vs 1-month follow-up test) and Group (training vs control) – p <0.001. The plot of the means is in Figure 4.

The “Test” variable is significantly changing from the pre-test to the 1-month follow-up test (increasing for the Training group, decreasing for the Control group) (p<0,05).

Differences between the training and control group (“Group” variable) also make a significant impact on the dependent variable “Test” (p<0,05).

***ANOVA results for the “Mental Health Awareness”***. Two independent factors (variables) were considered for the analysis with two levels for each of them – Test (pre-test vs 1-month follow-up test) and Group (training vs control). Aggregated measure *“Mental Health Awareness”* was assigned as the dependent variable. Results of the ANOVA are shown in Table 11.

**Table 11.** Mental Health Awareness (ANOVA results).

Source	Type III Sum of Squares	df	Mean Square	F	Sig.
Corrected Model	47.395 ^a^	3	15.798	13.162	<0.001
Intercept	30100.331	1	30100.331	25077.934	0.000
**Test (Pre, 1-month)**	17.780	1	17.780	14.813	<0.001
**Group (T, C)**	22.309	1	22.309	18.586	<0.001
Group * Test	5.976	1	5.976	4.979	0.026
Error	1073.043	894	1.200		
Total	31429.000	898			
Corrected Total	1120.438	897			

a) R Squared = 0.042 (Adjusted R Squared = 0.039)

**Figure 5.**
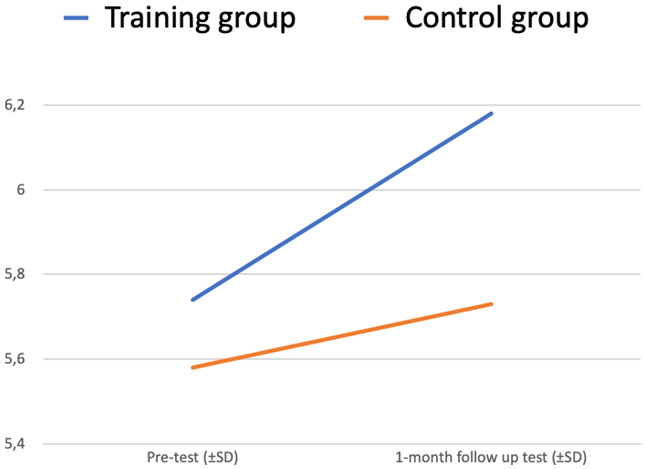
Mental Health Awareness (ANOVA plot).

There is a statistically significant interaction between two factors – Test (pre-test vs 1-month follow-up test) and Group (training vs control) – p <0.05. The plot of the means is in Figure 5.

The “Test” variable is significantly changing from the pre-test to the 1-month follow-up test (fast increasing for the Training group, slowly increasing for the Control group) (p <0.001).

Differences between the training and control group (“Group” variable) also make a significant impact on the dependent variable “Test” (p <0.05).

## Discussion

Analysis of changes over time in the training group received UMHT training revealed several significant changes related to the Readiness to face people with mental health issues at work and Mental health awareness. Absence of such changes over time in the control group that does not receive the UMHT support the hypothesis of UMHT participation as the cause of those changes.

*Readiness to face people with mental health issues at work* is increased significantly immediately after the training (effect size for each scale is in the range from middle to large). One month after the training, the cores slightly decreased, but the significance of the changes remained the same. One of the possible explanations for loosed scores between post-training and follow-up measurements is a reduction in the feeling of competence which was high immediately after training and went down in the following weeks. During post-test, the participants felt their readiness to face people with mental health issues because of intensive training of every needed skill and ongoing support from trainers who answered all the questions. A month later, the scores dropped due to apparent uncertainty when participants had to work alone without the continued support of mental health professionals (it is essential to mention that participants had the possibility of supervision during the month after training, but not everyone used it).

*Mental health awareness* scores demonstrate different tendencies. There is no significant increase in awareness immediately after the training. However, in one month, the differences are increasing, and mental health awareness scores are growing significantly to the middle level of the effect size. This delayed growth might be related to the knowledge application in practice. Mental health awareness is boosted after trainees have experienced a new way of interacting with people with mental health conditions in their workplace. The only scale that doesn’t change significantly over time is Attitudes toward people with mental health conditions. This is in line with explored earlier facts about the possibility of changing knowledge and behaviour more quickly than attitudes, which are well known as the most stable cognitions ( Livingston *et al*., 2013; Sampogna *et al*., 2017).

*Mental health proficiency* was explored only in the training groups. Mental health proficiency as proper recognition of mental disorders’ symptoms incised gradually in the UMHT from pre-test to follow-up test. Similar results were found in studies of similar programs. Evaluation of the Mental Health First Aid Training for the public shows rising numbers of depression and schizophrenia recognition among participants in pre-course, post-course, and six-month follow-up tests ( Kitchener & Jorm, 2002).

Proficiency is easy to measure, and usually, people are more likely to reproduce the information they heard than the actions they were trained in. It is because of a more complicated way of establishing a habit than remembering some facts. Thus, it was necessary to assess not just knowledge but actions and at least some aspects of the participant’s behaviour.

More than half of those professionals who participated in the trial (56,6%) admitted that they met people/clients with mental health conditions (primarily depression, panic disorders and autism spectrum disorder) and used obtained knowledge and skills in their routine work (67.7%). Among the most applicable skills are "paying attention to the person's condition" (90%), "getting ready for conversation" (69%), "building trust" (47%), "establishing contact" (45%), "preparing space" (44%), "bringing hope" (42%), "helping contact a professional" (42%), and "forming hypothesis" (41%) ( Figure 3).

Those preliminary data, in combination with the significant medium size effects related to increasing the readiness to interact with people with mental health issues at work, mental health awareness and mental health proficiency, are the arguments in favour of the effectiveness of the UMHT within low- and middle-income settings.

There are pieces of evidence of the effectiveness and feasibility of installing the task-shifting approach in low- and middle-income countries. The task-shifting results in decreasing stigma, strengthening referral pathways and increasing universal health coverage with a focus on equity of service provision ( Javadi *et al*., 2017; McInnis & Merajver, 2011; Scantlebury *et al*., 2018). The general framework for implementing this approach is the WHO Pyramid (Optimal Mix of Services) which implies that the vast majority of the population needs essential non-specialised mental health support ( WHO, 2003). Nonetheless, this is precisely the kind of support that is underdeveloped globally. To bridge the gap, the World Health Organization (WHO) presented the mhGAP Program, which includes the mhGAP Intervention Guide (for integrating mental health into the primary healthcare level) and a range of scalable psychological interventions to be delivered by non-medical professionals to people with mental health conditions ( WHO, 2008; WHO, 2016).

Although this is significant progress, those interventions are part of the formal health and social care system. Using them is demanding from professionals some amount of dedicated working time, and therefore not feasible to use them routinely without interrupting the main work processes. There is a need to develop scalable instruments for informal mental health support delivered in communities. As universal as possible, it is feasible to use in the routine work by a range of professionals who communicate with people and therefore have significant potential in addressing mental health issues: police workers, teachers, social workers etc.

The Mental Health First Aid seems to be such an instrument, but its implementation in the LMICs is challenging due to the system of dissemination ( Crisanti *et al*., 2015; Narayanasamy *et al*., 2018). Moreover, the main focus of the MHFA is the workplace, which makes it a perfect instrument for tackling stigma and increasing the level of mental health support within the organisation, but not for the mental health support that can be provided by frontline professionals to their clients, for example, while doing their primary job.

The Psychological First Aid, on the other hand, focuses on the immediate aftermath of crises and disasters ( WHO, 2011) but is unsuitable for people with manifested mental health conditions in the “normal” periods of life.

The Universal Mental Health Training, at the same time, combine all desirable feasibility options: it is developed within the LMICs for the LMICs; its steps are designed to be embedded in any working framework; therefore, using it is not time-consuming, and it is not interrupting the main workflow of any professional; it is explicitly developed to target mental health conditions that are relevant to a client’s profile, and it is flexible enough to allow tunning of the training content to needs of a particular group of professionals. Moreover, it is developed especially to be implemented for the range of non-health professionals while communicating with clients. Training in it is short and promisingly effective, as seen from the preliminary data.

### Limitation of the study

The study has its limitations. While training was delivered to the professionals of different fields, the data analysis was provided for the whole cohort of trainees without distinction between outcomes for types of professions. Only limited information on the participants was available, so age, gender, and working experience were not considered during the outcome measuring and data analysis. UMHT covers 18 mental health disorders, but in the pilot, not all conditions were included in the training program due to time constrictions. A follow-up assessment was made after the training, and in one month, an additional six-month follow-up is needed to understand the stability of the effect over time. Service users’ outcome measures were not applied in this study. Assessments of the TOT and supervisions were provided but not included in this study. Only questionnaires were used for the evaluation at this pre-piloting trial. The measurement methods used need further validation and reliability analysis.

## Conclusions

Increasing accessibility of mental health services and expanding universal health coverage is possible worldwide by using a task-shifting approach and delegating elements of mental health support to trained non-mental health service providers. The next delegation level is training and delivering parts of mental health support by non-healthcare workers and non-medical professionals in the communities. The main challenge of implementing this approach is that providing such support usually interrupts the professionals’ routine and standard workflow, distracting them from their primary tasks.

Developing an intervention that is at the same time effective, non-interrupting the working processes of the non-healthcare professionals and explicitly dedicated to the support of people with mental health conditions, and feasible for implementation in low- and middle-income countries is what we tried to achieve by constructing the Universal Mental Health Training.

Analysis of the outcome data revealed moderate effect size related to the knowledge of mental health conditions, mental health awareness, and increasing the readiness to interact with people with mental health issues at work, which consists of the readiness to face people with mental health issues at work, readiness to recognise MH condition, readiness to validate a condition with a person, readiness to give support, readiness to refer for professional help, readiness to ensure that professional service is received.

High-level utilisation of the UMHT at work by trained professionals confirms the effectiveness of the developed intervention. Results obtained favour the continuation of the development of the UMHT and future implementation research in this field in Ukraine and other low- and middle-income countries.

## Ethics and consent

All participants gave written informed consent to participate in the study. The research team adhered to the Declaration of Helsinki and Model Code of Ethics of the European Federation of Psychologists Associations (EFPA) and the Code of Ethics of the National Psychological Association of Ukraine, a member of the EFPA. The Ethics Committee of the Zhytomyr Ivan Franko State University approved the ethics protocol (registered in the Office for Human Research Protections), approval number 01–2905/2020 (29 May 2020).

## Data Availability

Zenodo: Underlying data for ‘Universal mental health training for frontline professionals: evaluation of pilot trial in Ukraine’, https://doi.org/10.5281/zenodo.10410525 ( Gorbunova & Klymchuk, 2023). The project contains the following underlying data: UMHT_dataset_pilot_trial.xlsx Data are available under the terms of the Creative Commons Zero “No rights reserved” data waiver (CC0 1.0 Public domain dedication).
