# Supplementary material for: Universal mental health training for frontline professionals: evaluation of pilot trial in Ukraine
Source: Open Res Eur. 2024 Jun 13;4:19. Originally published 2024 Jan 11. [Version 2] doi: 10.12688/openreseurope.16941.2 (PMC11249520; doi:10.12688/openreseurope.16941.2)
Supplement: Peer review history

# Reviewer report: Reviewer response for version 2

**DOI:** 10.21956/openreseurope.19501.r42077

**Published:** 2024-07-15

**Authors:** Monica-Georgiana Brinzac

**Affiliations:** 1 Babeș-Bolyai University, Cluj-Napoca, Romania

The article is thoroughly composed and offers a comprehensive overview of the research conducted. It effectively outlines the methodology, findings, and implications of the work, providing readers with a clear understanding of the study's scope and significance. The clarity and depth of the writing ensure that both the context and the details of the research are well communicated, making it a valuable resource for those interested in this area of study. The meticulous presentation reflects the robustness of the research process and the careful consideration of relevant factors, thereby enhancing the article's credibility and impact.

If applicable, is the statistical analysis and its interpretation appropriate?

Yes

Is the study design appropriate and is the work technically sound?

Yes

Is the work clearly and accurately presented and does it engage with the current literature?

Yes

Are the conclusions drawn adequately supported by the results?

Yes

Are sufficient details of methods and analysis provided to allow replication by others?

Yes

Are all the source data and materials underlying the results available?

Yes

Reviewer Expertise:

Workforce, medical deserts, youth, health systems, health policy

I confirm that I have read this submission and believe that I have an appropriate level of expertise to confirm that it is of an acceptable scientific standard.

# Reviewer report: Reviewer response for version 2

**DOI:** 10.21956/openreseurope.19501.r41442

**Published:** 2024-06-18

**Authors:** Joerg M Fegert, Anna Eberhardt

**Affiliations:** 1 University of Ulm, Ulm, Germany; 2 Department of Child and Adolescent Psychiatry/Psychotherapy, University of Ulm, Ulm, Baden-Württemberg, Germany; 3 Departement of Child and Adolescent Psychiatry/ Psychotherapy, University of Ulm, Ulm, Baden-Württemberg, Germany

I have no further comments to make.

If applicable, is the statistical analysis and its interpretation appropriate?

Yes

Is the study design appropriate and is the work technically sound?

Yes

Is the work clearly and accurately presented and does it engage with the current literature?

Yes

Are the conclusions drawn adequately supported by the results?

Yes

Are sufficient details of methods and analysis provided to allow replication by others?

Partly

Are all the source data and materials underlying the results available?

Yes

Reviewer Expertise:

Main areas of work include neglect, maltreatment, sexual abuse and other adverse childhood experiences as well as early intervention, mental health prevention, the relationship between youth welfare and child and adolescent psychiatry and other legal and forensic issues, focus on e-learning and dissemination, especially in the field of child protection and trauma research.

We confirm that we have read this submission and believe that we have an appropriate level of expertise to confirm that it is of an acceptable scientific standard.

# Reviewer report: Reviewer response for version 1

**DOI:** 10.21956/openreseurope.18308.r40131

**Published:** 2024-05-16

**Authors:** Joerg M Fegert, Anna Maier

**Affiliations:** 1 University of Ulm, Ulm, Germany; 2 Department of Child and Adolescent Psychiatry/Psychotherapy, University of Ulm, Ulm, Baden-Württemberg, Germany

**Introduction:**

- The introduction is logically structured and leads from the general challenges to the specific intervention analyzed.
- However, some of the language could be simplified for readers who are not familiar with the psychological/Ukrainian context. For example, complex terms such as "task-shifting approach" could be defined or briefly explained to facilitate understanding.
- Some claims lack direct references to specific studies. Providing more direct references would strengthen the academic rigors of the manuscript and facilitate further exploration by readers.
- TOT approaches should be described in more detail in terms of procedure and evidence.

**Methods:**

- The section is well structured with clear information on participant demographics, training modules, outcome assessments and methods of data analysis.
- Information on any incentives or motivations for participation would improve understanding.
- Further details on the data collection process, including how responses were collected and the measures taken to ensure data quality are necessary.
- Information on ethical approval and any measures taken to ensure participant confidentiality is missing from the methods section.

**Results:**

- The manuscript contains a comprehensive analysis of the impact of mental health training on professionals' readiness to deal with people with mental health problems and on their general awareness of mental health.
- A brief description of the methods used to calculate the effect size and its significance would improve the understanding of the results.
- Some figures lack detailed labelling, which makes it difficult to interpret the information.

**Discussion:**

- Discussion of possible strategies to address the identified shortcomings and problems. This would further improve the manuscript's contribution to the field of global mental health.

If applicable, is the statistical analysis and its interpretation appropriate?

Yes

Is the study design appropriate and is the work technically sound?

Yes

Is the work clearly and accurately presented and does it engage with the current literature?

Yes

Are the conclusions drawn adequately supported by the results?

Yes

Are sufficient details of methods and analysis provided to allow replication by others?

Partly

Are all the source data and materials underlying the results available?

Yes

Reviewer Expertise:

Main areas of work include neglect, maltreatment, sexual abuse and other adverse childhood experiences as well as early intervention, mental health prevention, the relationship between youth welfare and child and adolescent psychiatry and other legal and forensic issues, focus on e-learning and dissemination, especially in the field of child protection and trauma research.

We confirm that we have read this submission and believe that we have an appropriate level of expertise to confirm that it is of an acceptable scientific standard, however we have significant reservations, as outlined above.

# Response

**Published:** 2024-06-07

**Authors:** Viktoriia Gorbunova

**Affiliations:** Department of Behavioural and Cognitive Sciences, University of Luxembourg, Esch-sur-Alzette, Luxembourg District, Luxembourg

Dear colleagues, 

 Thank you for reviewing our work and for your valuable feedback. Please see our answers below and consider that all mentioned changes will be provided in a new version of the article.

 •    However, some of the language could be simplified for readers who are not familiar with the psychological/Ukrainian context. For example, complex terms such as "task-shifting approach" could be defined or briefly explained to facilitate understanding.

 An extended definition is added to the abstract.

 •    TOT approaches should be described in more detail in terms of procedure and evidence. An extended definition is added to the first mention of the approach.

 •    Information on any incentives or motivations for participation would improve understanding. The sentence about the absence of any incentives is added to the relevant section.

 •    Further details on the data collection process, including how responses were collected and the measures taken to ensure data quality, are necessary. The section on data collection has been created, and necessary details have been added. 

 •    Information on ethical approval and any measures taken to ensure participant confidentiality is missing from the methods section. The relevant section is positioned at the end of the article due to the Open Science requirements. 

 •    A brief description of the methods used to calculate the effect size and its significance would improve the understanding of the results. It is stated in the Method section, Data analysis sub-section, that “Student’s T-test, dependent samples, was used to analyse the statistical significance of changes before and after the training and one month after. Cohen’s effect size coefficients were calculated to estimate the size of the effect. Two-way ANOVA was utilised to analyse the significance of differences for aggregated indexes in training and control groups before and one month after the training.”

 •    Some figures lack detailed labelling, which makes it difficult to interpret the information. We are sorry for the confusion; it might be due to an unclear figure description. It is corrected for Figures 2 and 3.

# Reviewer report: Reviewer response for version 1

**DOI:** 10.21956/openreseurope.18308.r39598

**Published:** 2024-05-08

**Authors:** Roberto Mediavilla

**Affiliations:** 1 Department of Psychiatry, Universidad Autonoma de Madrid, Madrid, Community of Madrid, Spain

In this study, the authors present the results of a trial comparing the effects of a mental health training programme for frontline workers in Ukraine, across a series of outcomes related to mental health. The findings show differences between the two groups which could potentially be caused by treatment allocation. However, major limitations, including unsuccessful randomisation, use of non-validated self-reports, and unadjusted models, significantly hamper the interpretation of the results.

**Abstract**

- “Controlled pilot trial of X’s efficacy”. I am not sure about this. You mention efficacy here for the first time, but you do not mention any specific outcome.
- What is “a five-step model” of mental health?
- “and the final module that considers possible difficulties and ways of their solving”. I don’t understand.
- “Three efficacy measurements were used in the outcome assessment: Readiness to interact with people with mental health issues at work, mental health awareness and mental health proficiency”. And how were they assessed? What was the primary outcome measure?
- “middle-level effect size”. Do you mean medium/moderate effect sizes?
- “Analysis of the outcome data revealed middle-level effect size related to the knowledge of mental health conditions, mental health awareness, and increasing the readiness to interact with people with mental health issues at work in first-line professionals that underwent the UMHT.” And in the other arm? Efficacy/Effectiveness always involves comparators.
- “High-level utilisation of the UMHT at work by trained professionals confirms our hypothesis on the efficacy and feasibility of the developed intervention.” Which were your hypotheses? You did not mention anything about feasibility (nor have you any outcomes that let you talk about feasibility, at least not in the Abstract)
- “Results obtained favour the continuation of the development of the UMHT and future implementation research in this field in Ukraine and potentially in other low- and middle-income countries.” This cannot be extracted from the Abstract, at least not as it reads right now

**Introduction**

- “Ukraine is a country from the post-Soviet space where psychiatry was a punishment instrument of the regime rather than a health care service (Weissbecker et al., 2017).” This is not supported by Weissbecker et al. 2017, which provides policy recommendations but was not designed to test such hypothesis.
- Please define “task-shifting approach in mental health”
- “Nonetheless, there is a lack of universality (as in the case of the mhGAP), scalability (e.g. local programmes for frontline workers), and feasibility for the LMICs (e.g. the MHFA)”. This sentence seems to be your rationale for conducting the study but I find it extremely vague. I suggest you spend some time in reinforcing the references and pointing at the main research gap that this research will fill.
- “Frontline non-health professionals”. The term frontline implies some sort of crisis, at least to me -I am not a native English speaker. When you say frontline workers, you mean those providing support to communities affected by the war in Ukraine? You may need to specify if that is the case.
- “This paper aims to analyse the results of piloting the Universal Mental Health Training (UMHT) implementation.” This objective is very vague. In this paragraph, you should state how you will fill the research gap. You should present your objective (at least the primary aim) and then briefly say how you would achieve it (e.g., in your case, you will use an RCT design).
- Since this is an RCT, and the UMHT is your intervention, you should describe it in the Methods section. You could mention it in the Introduction, as part of your rationale (in fact, you must), but the full description should be in Methods and potentially in the Appendix if it is too long.

**Methods**

- Start with a full description of the study design before mentioning the participants.
- “The controlled pilot trial of UMHTs’ efficacy was conducted with 307 frontline professionals divided into 24 training groups (social workers (12 groups, 128 persons), educators (4, 63), police officers (4, 60), priests and clerics (1, 15), military volunteers (1, 12), workers of occupation centres (1, 13), emergency workers (1, 16)).” This is not a standard way of reporting frequencies. Consider providing the average number of people per group and provide the details in the Appendix.
- “All participants were recruited for training by their team leaders, who were informed about training possibilities by letters sent from the training developers. The only requirement for participation was working in the field with people.” Which team leaders? Of which teams? Which training developers? What do you mean by “working in the field with people”?
- A primary endpoint is missing -and so is any reference to primary outcomes or timepoints.
- It seems that all measures were developed ad hoc for this study, is that right? That is a major limitation, especially if you rely on self-reports only.
- “Two-way ANOVA was utilised to analyse the significance of differences for aggregated indexes in training and control groups before and one month after the training.” What does “aggregated indexes” mean? If you are estimating the group*time interaction (which makes sense considering the study design), why do you perform two additional T-tests? If the study is sufficiently powered (something the reader doesn’t know at this point), any baseline differences will be due to chance, hence the independent-samples T-test is useless. The dependent-samples T-test is also redundant with the time effects estimated by the ANOVA model.
- I find the study design at the end of the Methods section, which is a bit confusing. I also find out here that the study is not randomised “because it was not possible”. Why is that? What is then your study design? If this is an observational study, because participants were not randomised, you should probably re-think your data analysis plan. Your models should be adjusted for potential confounding if you want to make any claim regarding the effect of the exposure on the outcomes.

Please revise this section and think that your study has to be fully replicated using the information provided here. Please follow the CONSORT guidelines for RCTs and kindly provide the CONSORT checklist as a supplement. That will help cover all relevant aspects of it. It will also help the reader better understand the study.

**Results**

 My main concern here is that (a) you did not randomised your study participants and (b) there seems to be large differences between the groups at baseline (Table 3). If you do not control for baseline differences, how would you make any statements about the effect of your program?

 This adds to the fact that (a) you have not pre-specified a primary outcome measure and (b) you have several outcomes (on which you conduct three different tests, including two Students T and one ANOVA). This makes the results very hard to follow and your main point gets blurred.

**Discussion**

 I have not revised the Discussion section due to the major limitations that I extracted from the study design, data analysis plan, and results.

**General points**

- I think the manuscript could benefit from some language-editing services

**Minor points**

- Abstract. What are frontline professionals? Please define
- Explain acronyms (e.g., WHO, mhGAP)

If applicable, is the statistical analysis and its interpretation appropriate?

No

Is the study design appropriate and is the work technically sound?

No

Is the work clearly and accurately presented and does it engage with the current literature?

Partly

Are the conclusions drawn adequately supported by the results?

No

Are sufficient details of methods and analysis provided to allow replication by others?

No

Are all the source data and materials underlying the results available?

Yes

Reviewer Expertise:

Clinical trials, mental health, psychological interventions, anxiety, epression

I confirm that I have read this submission and believe that I have an appropriate level of expertise to state that I do not consider it to be of an acceptable scientific standard, for reasons outlined above.

# Response

**Published:** 2024-05-13

**Authors:** Viktoriia Gorbunova

**Affiliations:** Department of Behavioural and Cognitive Sciences, University of Luxembourg, Esch-sur-Alzette, Luxembourg District, Luxembourg

Dear Roberto Mediavilla, Thank you for the review of our work. First and foremost, we want to point out that we never stated that our study is RCT.  As is written in the Study Design section, the first sentence: “The study was quasi-experimental (no complete randomization was possible at this piloting stage). Two groups were involved, the experimental group (received UMHT) and the control group (no training, waiting list). The complete outline of the study design is in Table 2.” There are various experimental controlled studies besides RCTs, which are commonly used, especially in implementation research and pilot studies in real-life circumstances outside the laboratory conditions.

 We hope that the fact that our study is not an RCT resolves the majority of your concerns about its compliance with the RCT standards. That is also the main reason why we didn’t use the CONSORT reporting standards. However, we acknowledge the potentially misleading wording in the Method section. We used “controlled trial” that is commonly (but not only) used in clinical reseacrh and RCTs descriptions. In the next version of the article, which will be developed after receiving all necessary reviews, we will change it to the word “study”.  

*Answers to the remarks and recommendations for the Abstract section.*

  

- “Controlled pilot trial of X’s efficacy”. I am not sure about this. You mention efficacy here for the first time, but you do not mention any specific outcome.

There is a discussion about using the terms "efficacy" and "effectiveness" in assessing different interventions. Even though many authors use the words interchangeably, we agree that the term "effectiveness" would better suit our research as it reflects intervention performance in real-life circumstances. We will change it in the next article iteration. Regarding your remark about the absence of the outcomes - they are mentioned in the 4th sentence of the Abstract section you are referring to. Namely: "Three efficacy measurements were used in the outcome assessment: Readiness to interact with people with mental health issues at work, mental health awareness and mental health proficiency." Full descriptions of the outcomes and measures are provided in the relevant section of the article (see Methods).

- What is “a five-step model” of mental health?

We do not mention the "five-step model of mental health". We write, "All the groups took part in eight-hour training which includes one introductory module that introduces the mental health topic alongside a five-step model, two disorders-focused modules with the steps adjusted to work with specific disorders and the final module that considers possible difficulties and ways of their solving." By the five-step model, we meant the five-step model of UMHT, Universal Mental Health Training. It is described in detail in the section "Universal Mental Health Training (UMHT): a new instrument for informal mental health response by frontline professionals." To make it clearer, we will add in the abstract: "a five-step model of Universal Mental Health Training."

- “and the final module that considers possible difficulties and ways of their solving”. I don’t understand.

As the training is about interacting with people with mental health conditions and is devoted to training people with no prior experience in mental health support, many questions and problems arise during such communication. So, this module serves to prevent them and to help professionals be prepared to solve such communication and interaction problems. We will extend the explanation in the abstract and adjust the text.

- Three efficacy measurements were used in the outcome assessment: Readiness to interact with people with mental health issues at work, mental health awareness and mental health proficiency”. And how were they assessed? What was the primary outcome measure?

The relevant section of the article describes measurement in detail (see Methods, sub-section Measurement).

- “middle-level effect size”. Do you mean medium/moderate effect sizes?

To the best of our knowledge, the terms are equivalent. However, your wording is more common, so changing the words is no problem.

- “Analysis of the outcome data revealed middle-level effect size related to the knowledge of mental health conditions, mental health awareness, and increasing the readiness to interact with people with mental health issues at work in first-line professionals that underwent the UMHT.” And in the other arm? Efficacy/Effectiveness always involves comparators.

There were no effects in the control group, so we didn’t mention them in the abstract. All the information is available in the relevant section of the article (see Results). However, we will add the sentence about the absence of changes in the control group to the abstract.

- “High-level utilisation of the UMHT at work by trained professionals confirms our hypothesis on the efficacy and feasibility of the developed intervention.” Which were your hypotheses? You did not mention anything about feasibility (nor have you any outcomes that let you talk about feasibility, at least not in the Abstract)

We can only agree that the wording is not correct. Initially, this article was part of a larger text about the pilot and feasibility study, and this wording has to be changed.

- “Results obtained favour the continuation of the development of the UMHT and future implementation research in this field in Ukraine and potentially in other low- and middle-income countries.” This cannot be extracted from the Abstract, at least not as it reads right now

We see that in this way: if the pilot trial produces some positive results, it supports the idea of further development, research, and dissemination. We would write differently if the results were negative or more neutral.  

*Answers for the remarks and recommendations for the Introduction section.*

- “Ukraine is a country from the post-Soviet space where psychiatry was a punishment instrument of the regime rather than a health care service (Weissbecker et al., 2017).” This is not supported by Weissbecker et al. 2017, which provides policy recommendations but was not designed to test such hypothesis.

That is not a hypothesis; that is something we (Ukrainians) were born to and lived through. See p. 84 of the reference provided (Weissbecker et al., 2017). Additional reference will be provided in the second version of the article: MHPSS in Ukraine | ARQ

- Please define “task-shifting approach in mental health”

It is defined with the reference on the article's first page (see Introduction): “The task-shifting approach in mental health as a partial distribution of care to frontline professionals seems beneficial for persons with mental health issues and the system itself. It was proven that task shifting in the mental health area eases the workload of health professionals and strengthens community mental health systems, which are essential for low- and middle-income countries ( Javadi *et al.,* 2017; McInnis & Merajver, 2011).”

- Nonetheless, there is a lack of universality (as in the case of the mhGAP), scalability (e.g. local programmes for frontline workers), and feasibility for the LMICs (e.g. the MHFA)”. This sentence seems to be your rationale for conducting the study but I find it extremely vague. I suggest you spend some time in reinforcing the references and pointing at the main research gap that this research will fill.

The mentioned programmes were developed and rigorously studied, with many systematic reviews and meta-analyses. The aim of the article was not to dive deep into the analysis that had already been done but to describe our ideas and the pilot study results. Besides, the article is focused on specialists who already know about such programmes, task-shifting approach, and global mental health in general. However, we discuss the mentioned programmes in the relevant section of the article (see Discussion). It is a pity you consciously decided not to read the Discussion section, as many points are highlighted and expanded there.

- “Frontline non-health professionals”. The term frontline implies some sort of crisis, at least to me -I am not a native English speaker. When you say frontline workers, you mean those providing support to communities affected by the war in Ukraine? You may need to specify if that is the case.

There were 27 times we mentioned “frontline professionals” or “frontline workers” and only one “frontline non-health professionals”. We should admit that health professionals can be frontline workers; however, in our study, they were not represented, which is why we used such wording once (we will remove it in the next version of the article). 

 As for the definition "frontline professionals", it is provided in the relevant part of the article (see Introduction): “The target audience for the UMHT delivery is frontline professionals (workers), defined as professionals whose jobs involve close personal communication with people (clients, service users, etc.) (Blau et al., 2021). The most common types of frontline professionals have been identified according to Ukrainian occupational regulations and standards: social workers, educators, police officers, priests and clerics, military volunteers, workers of occupation centres, emergency workers, etc. ( Derzhspozhyvstandart of Ukraine, 2010).” The term is commonly used and has no relation to the war, despite the fact that a lot of the training participants were located in the East of Ukraine, in the regions affected by the war (before the full-scale invasion). See Blau *et al*., 2021.

 Also, we use the term "first-line professionals" as a synonym for "frontline professionals" two times in the article, but as there is no definition, we will change it to "frontline professionals".

- “This paper aims to analyse the results of piloting the Universal Mental Health Training (UMHT) implementation.” This objective is very vague. In this paragraph, you should state how you will fill the research gap. You should present your objective (at least the primary aim) and then briefly say how you would achieve it (e.g., in your case, you will use an RCT design).

Thank you for your valuable input. We will elaborate more on this part of the article.

- Since this is an RCT, and the UMHT is your intervention, you should describe it in the Methods section. You could mention it in the Introduction, as part of your rationale (in fact, you must), but the full description should be in Methods and potentially in the Appendix if it is too long.

We didn’t use RCT as was explained above.  

*Answers for the remarks and recommendations for the Methods section.*

- Start with a full description of the study design before mentioning the participants.

There are different ways of structuring the Method section. We are not sure that one way is better than another. We are psychologists by background; that is why, for us, people always come first. Therefore, we use such an approach in many other articles. No proposals like yours have been delivered to us previously, but that is something to consider in the future.

- “The controlled pilot trial of UMHTs’ efficacy was conducted with 307 frontline professionals divided into 24 training groups (social workers (12 groups, 128 persons), educators (4, 63), police officers (4, 60), priests and clerics (1, 15), military volunteers (1, 12), workers of occupation centres (1, 13), emergency workers (1, 16)).” This is not a standard way of reporting frequencies. Consider providing the average number of people per group and provide the details in the Appendix.

The underlying data file (see  Zenodo (Gorbunova & Klymchuk, 2023)) provides all the details; information about the number of participants in each group can be extracted from there. As we didn’t describe the group-to-group comparison in the article (it was not our subject of exploration in this case), we do not see the need to overload the text with additional numbers that will not add value to the article.

- “All participants were recruited for training by their team leaders, who were informed about training possibilities by letters sent from the training developers. The only requirement for participation was working in the field with people.” Which team leaders? Of which teams? Which training developers? What do you mean by “working in the field with people”?

Which team leaders? – our training participants work in different services and have leaders of the teams (general terms for the chief, director, etc.). Of which teams? – teams in which our participants work. Which training developers? – we, the authors of the article, as described earlier in the relevant section of the article. What do you mean by “working in the field with people”? – exactly what is written; we used “field” as a commonly used term for the work area where professionals deliver their services for clients/service users, etc.

- A primary endpoint is missing -and so is any reference to primary outcomes or timepoints.

Sorry, we do not understand the comment.

- It seems that all measures were developed ad hoc for this study, is that right? That is a major limitation, especially if you rely on self-reports only.

It is mentioned in our Limitation section. The relevant section (see Methods) provides a detailed description of all the measures.

- “Two-way ANOVA was utilised to analyse the significance of differences for aggregated indexes in training and control groups before and one month after the training.” What does “aggregated indexes” mean? If you are estimating the group*time interaction (which makes sense considering the study design), why do you perform two additional T-tests? If the study is sufficiently powered (something the reader doesn’t know at this point), any baseline differences will be due to chance, hence the independent-samples T-test is useless. The dependent-samples T-test is also redundant with the time effects estimated by the ANOVA model.

Aggregated indexes are described in the section Results of the ANOVA for the aggregated indexes. In general, we agree; however, the ANOVA allowed us to see the differences better and have a better picture of the study results. Also, it was due to the nonequivalence of the baselines in the experimental and control groups.

- I find the study design at the end of the Methods section, which is a bit confusing. I also find out here that the study is not randomised “because it was not possible”. Why is that? What is then your study design? If this is an observational study, because participants were not randomised, you should probably re-think your data analysis plan. Your models should be adjusted for potential confounding if you want to make any claim regarding the effect of the exposure on the outcomes.

Randomisation was impossible due to the study's implementation character, real-life restrictions, and other circumstances (budget limitation, for example). Different experimental study designs exist and are widely used, and our study is not an RCT, as described in the Method section and explained above in this scope of responses. That is why we additionally use ANOVA.

- Please revise this section and think that your study has to be fully replicated using the information provided here. Please follow the CONSORT guidelines for RCTs and kindly provide the CONSORT checklist as a supplement. That will help cover all relevant aspects of it. It will also help the reader better understand the study.

All our data are in the Open Access. Our study was not an RCT, and replicating the pilot implementational studies is quite a questionable task. The CONSORT is not used for non-RCT non-clinical studies.  

*Answers for the remarks and recommendations for the Results section.*

- My main concern here is that (a) you did not randomised your study participants and (b) there seems to be large differences between the groups at baseline (Table 3). If you do not control for baseline differences, how would you make any statements about the effect of your program?
  This adds to the fact that (a) you have not pre-specified a primary outcome measure and (b) you have several outcomes (on which you conduct three different tests, including two Students T and one ANOVA). This makes the results very hard to follow and your main point gets blurred.

Again, our study is not an RCT; it is a pilot implementation study with a quasi-experimental design. There are definitely limitations to this study, and therefore, we conducted additional statistical analysis, exploring the dataset from different perspectives. All the limitations are mentioned in the relevant section of the article, which is after the Discussion that you didn’t read.  

*Discussion section.*

- I have not revised the Discussion section due to the major limitations that I extracted from the study design, data analysis plan, and results.

That is a pity, as it is questionable how one can review the article and make any decision about it without reviewing all of the text. This section would probably provide answers to some of the questions raised.  

*Minor points.*

- Abstract. What are frontline professionals? Please define
- Explain acronyms (e.g., WHO, mhGAP)

As for the abstract, we are not sure that the definition of the commonly used term must be included in the abstract, which is dedicated to the short description of the study results. The definition is provided in the relevant section of the article. WHO—World Health Organisation; this is the first time we have been asked to explain WHO's acronym. mhGAP—Mental Health Gap Action Programme; agree. A non-knowledgeable audience might be confused by this abbreviation, so we will explain it at the first mention.
